# Intraamniotic Administration (*Gallus gallus*) of Genistein Alters Mineral Transport, Intestinal Morphology, and Gut Microbiota

**DOI:** 10.3390/nu14173473

**Published:** 2022-08-24

**Authors:** Jacquelyn Cheng, Nikolai Kolba, Philip Sisser, Sondra Turjeman, Carmel Even, Omry Koren, Elad Tako

**Affiliations:** 1 Department of Food Science, Cornell University, Stocking Hall, Ithaca, NY 14853, USA; 2 Department of Environment & Sustainability, Cornell University, Kennedy Hall, Ithaca, NY 14853, USA; 3 Azrieli Faculty of Medicine, Bar-Ilan University, Safed 1311502, Israel

**Keywords:** genistein, brush border membrane, iron, microbiome, biomarkers

## Abstract

Genistein is an isoflavone naturally present in numerous staple food crops, such as soybeans and chickpeas. This study utilized the *Gallus gallus* intraamniotic administration procedure to assess genistein administration effects on trace mineral status, brush border membrane (BBM) functionality, intestinal morphology, and intestinal microbiome in vivo. Eggs were divided into five groups with 1 mL injection of the following treatments: no-injection, DI H_2_O, 5% inulin, and 1.25% and 2.5% genistein (*n* = 8 per group). Upon hatch, blood, cecum, small intestine, and liver were collected for assessment of hemoglobin, intestinal microflora alterations, intestinal morphometric assessment, and mRNA gene expression of relevant iron and zinc transporter proteins, respectively. This study demonstrated that intraamniotic administration of 2.5% genistein increased villus surface area, number of acidic goblet cells, and hemoglobin. Additionally, genistein exposure downregulated duodenal cytochrome B (DcytB) and upregulated hepcidin expression. Further, genistein exposure positively altered the composition and function of the intestinal microbiota. Our results suggest a physiological role for genistein administration in improving mineral status, favorably altering BBM functionality and development, positively modulating the intestinal microbiome, as well as improving physiological status.

## 1. Introduction

Genistein is a polyphenolic isoflavone naturally found in numerous staple crops, including soybeans and chickpeas. Many studies have reported genistein to possess various beneficial and protective physiological properties, with effects observed in metabolic syndrome, diabetes, and breast and prostate cancers in vivo [1,2]. The biological effects of isoflavone consumption have been attributed to structural similarity and function with human and animal estrogens. Specifically, due to structural similarity to 17b-estradiol, genistein has been observed to possess weak estrogenic activity and exhibit preferential binding to estrogen receptor ß [2,3].

The characterization of genistein metabolism and absorption is still ongoing, despite the well-studied physiological effects of genistein and other isoflavones. Dietary isoflavones exist as isoflavone-glycosides and are transformed by intestinal microbiota via bacterial enzymatic action to more potent metabolites, such as equol and O-desmethylan- lensin [4]. Thus, individual differences in gut microbiota will consequently be expected to influence the potential for physiological effects associated with isoflavone ingestion [5]. Current research has shown genistein administration in mice fed a high-fat diet ameliorated harmful effects associated with a high-fat diet through increasing populations of bacteria associated with reduced pro-inflammatory lipopolysaccharide and lower serum triglyceride levels [1]. Another recent study has shown that isoflavone administration in vitro promoted short-chain fatty acid (SCFA) production due to increased proliferation of SCFA-producing bacteria species from *Clostridium* cluster XIVa, *Roseburia* and *E. hallii* [4]. Additionally, maternal genistein intake perinatally and throughout pregnancy in mice mitigated harmful effects of a high-fat fed diet in dams and offspring and was associated with an increase in butyrate-producing gut bacteria [6]. Increased SCFA production has been associated with inhibiting harmful pathogen growth, decreased intestinal pH, and upregulated brush border membrane (BBM) gene expression [7,8]. Taken together, these effects enhance micronutrient bioavailability.

Emerging evidence suggests that genistein exposure could be implicated in the altered expression of proteins involved in iron (Fe) transport. Genistein significantly increased Fe export through estrogen receptor ß-dependent p38 MAPK up-regulation through ceruloplasmin and ferroportin-1 in glial cells [9]. However, another study found that genistein treatment of human hepatocytes increased both hepcidin transcription levels and promoter activity (hepcidin decreases intestinal Fe absorption by inhibiting ferroportin) [10].

Despite the investigation of specific health benefits attributed to dietary genistein administration and subsequent knowledge of genistein ingestion on gut microbiota modulation and Fe transport, there is a paucity of knowledge regarding how genistein affects the brush border membrane (BBM) of the small intestine. As BBM functional capacity (i.e., digestive enzyme production) dictates the extent of food digestion and absorption, it is key to investigate the interactions between bioactive compounds in the diet and the BBM. There is also a lack of studies that specifically utilize the embryonic stage of the *Gallus gallus* for elucidating the effects of genistein consumption on BBM development and functionality. Due to similarities in intestinal morphology, microbiota, and gene homology of duodenal mineral transporters between humans and *Gallus gallus*, the *Gallus gallus* has been used as a novel and cost-effective animal model to elucidate the physiological effects of plant bioactives and nutritional solutions relevant to human nutrition [11,12,13,14,15]. To study the impact of bioactive on the embryonic stage, the intraamniotic administration approach can be utilized for testing the effects of the solution administered into the amniotic fluid on the different systems of interest in a closed system, where the amniotic fluid is naturally and orally consumed by the embryo starting at day 17 and is entirely consumed by hatch [7,11,16,17,18].

In our present study, the effects of genistein intraamniotic administration on brush border membrane (BBM) functionality, intestinal morphology, and intestinal microbiome were studied in vivo using the embryonic stage of the *Gallus gallus*. It was previously demonstrated that daidzein, another major isoflavone found in soybeans with estrogenic effects, altered BBM Fe transport proteins and cecal bacterial populations in the embryonic stage of the *Gallus gallus* [19]. Therefore, the first objective of this study was to evaluate genistein administration effects on BBM functionality through evaluating duodenal gene expression of biomarkers of mineral status, BBM digestive and absorptive ability, and inflammation. To accomplish this objective, we assessed the expression of duodenal cytochrome B (DcytB, a Fe-specific cytochrome reductase on the luminal side of the enterocyte) and divalent metal transporter 1 (DMT1, the primary transporter of Fe^2+^ from the luminal side of the enterocyte), ferroportin (a basolateral exporter of dietary Fe^2+^), liver hepcidin (decreases intestinal Fe absorption by inhibiting ferroportin), as well as duodenal ZnT7 (zinc transporter protein 7) and ZIP6 (zinc transporter) [10]. BBM digestive and absorptive ability were evaluated by assessing duodenum morphology and gene expression of biomarkers of BBM digestive and absorptive ability (AP—aminopeptidase, SI—sucrase-isomaltase, and NaK/ATPase—sodium-potassium adenosine triphosphatase). In addition, systemic inflammatory status was evaluated using the expression of immunoregulatory cytokines (TNF-α, tumor necrosis factor-alpha; and NF-κB, nuclear factor kappa B subunit 1). The second objective was to utilize PCR quantification to analyze duodenal microbial populations and next-generation sequencing to analyze the cecal microbiome to elucidate potential alterations in gut microbiota composition and function resulting from genistein administration. We hypothesize that when administered intraamniotically, genistein will alter mineral transport, cause favorable alterations in BBM functionality and development, and positively modulate the gut microbiota.

## 2. Materials and Methods

### 2.1. Animals and Experimental Design

Fertile Cornish-cross broiler eggs (*Gallus gallus*) were acquired (Moyer’s chicks, Quakertown, PA, USA) and incubated utilizing optimum conditions at the Cornell University Animal Science Poultry Farm Incubator [20]. The protocol was approved by the Cornell University Institutional Animal Care and Use Committee (IACUC #2020-0077). On incubation day 17, viable embryos were weighed, and eggs were randomly distributed by weight into five groups (*n* = 8 per group, each group contained eggs of similar weight frequency distribution). Treatments in powder form were prepared in DI H_2_O. The experimental groups were as follows: two treatment groups (1.25, 2.5% genistein), two controls (H_2_O injection and no-injection), and a positive control (5% inulin). After identification of the injection site via candling, 1 mL of experimental solution was injected into the amniotic fluid of each egg using a 21-gauge needle. After injection, the injection holes were sterilized with 70% ethanol and sealed. Eggs were returned to the incubator with equal representation at each incubator location to reduce allocation bias. Immediately upon hatch (day 21), blood was collected, and all chicks were euthanized by CO_2_ exposure. The small intestine, cecum, pectoral muscle, and liver were collected, placed in liquid nitrogen for immediate freezing, and stored at −80 °C until analysis.

### 2.2. Blood Hemoglobin (Hb) Measurements

Blood was collected in sodium heparin tubes (ThermoFisher Scientific, Waltham, MA, USA). The QuantiChrom^TM^ Hemoglobin Assay (BioAssay Systems, Hayward, CA, USA) was utilized to quantify hemoglobin (Hb) concentrations spectrophotometrically following the manufacturer’s instructions.

### 2.3. Total RNA Isolation from Duodenum and Liver Tissue Samples

Total RNA was extracted from 30 mg of duodenal (*n* = 6) or liver tissues (*n* = 6) according to the manufacturer’s instructions under RNase-free conditions using the Qiagen RNeasy Mini Kit (Qiagen Inc., Valencia, CA, USA). RNA was quantified by the ratio of absorbance (260/280 nm) using a NanoDrop 2000 (ThermoFisher Scientific, Waltham, MA, USA). RNA samples were stored at −80 °C until use.

### 2.4. Real-Time Polymerase Chain Reaction (RT-PCR)

As was previously described [12,16,17,21], cDNA was made using a 20uL reverse transcriptase (RT) reaction in a BioRad C1000 Touch Thermal Cycler using the Improm-II Reverse Transcriptase Kit (Promega, Madison, WI, USA). The reverse transcriptase reaction consisted of the following: 1 μL total RNA template, 10 μM random hexanucleotide primers, and 2 mM of oligo(dT) primers. Reactions were completed in conditions as indicated: 94 °C for 5 min, 60 min at 42 °C, 70 °C for 15 min, and hold at 4 °C. cDNA concentration was determined using a NanoDrop 2000 (ThermoFisher Scientific, Waltham, MA, USA) by measuring the ratio of absorbance (260/280 nm).

#### 2.4.1. Primer Design

As was previously described [12,16,17,21], primers were designed using the PrimerQuest Tool (IDT DNA, Coralvilla, IA, USA) based on 13 genetic sequences publicly available on the GenBank database. DNA sequences of primers utilized in this study are summarized in Table 1.

#### 2.4.2. Real-Time qPCR Design

RT-qPCR was performed as was previously described [12,16,17,21]. Briefly, 10 μL RT-qPCR reactions comprised cDNA, SYBR Green Supermix (2X BioRad SSO Advanced Universal, Cat #1725274, Hercules, CA, USA), forward and reverse primers (as shown in Table 1), and nuclease-free H_2_O. DNA amplification was performed under the following conditions: first denaturation at 95 °C for 30 s, 40 cycles of denaturation at 95 °C for 15 s, various annealing temperatures based on the primers utilized (PrimerQuest Tool, IDT DNA, Coralvilla, IA, USA) for 30 s and elongation at 60 °C for 30 s using a Bio-Rad CFX96 Touch (Hercules, CA, USA). Cp values were calculated using the automated “second derivative maximum” method (Bio-Rad CFX Maestro Software Version 4.1.2433.1219, Hercules, CA, USA). Gene expression was normalized to 18S gene expression [22]. RT-qPCR efficiency values for the 13 genes were as follows: DcytB, 1.046; DMT 1, 0.998; Ferroportin, 1.109; Hepcidin, 0.976; Δ-6-Desaturase, 0.925; ZIP6, 0.961; ZnT7, 0.916; NK-κβ, 1.113; TNF-α, 1.046; AP, 1.015; SI, 1.032; NaK/ATPase, 1.024; and 18S rRNA, 0.994.

### 2.5. Collection of Microbial Samples and Intestinal Contents DNA Isolation

As was previously described [16,21], intestinal contents were placed into a sterile 15 mL tube (Corning, Corning, NY, USA), 9 mL 1X phosphate buffered saline (PBS) was added, and the contents were vortexed with silicone beads (3 mm) for 3 min and centrifuged at 1000× *g* for 5 min. The supernatant was collected and centrifuged at 4000× *g* for 20 min, and the resulting pellet was washed twice with PBS. The pellet was dissolved in 50 mM EDTA and incubated with 10 mg/mL lysozyme (Sigma Aldrich CO., St. Louis, MO, USA) for 45 min at 37 °C. A Wizard Genomic DNA purification kit (Promega Corp., Madison, WI, USA) was used to isolate bacterial genomic DNA according to the manufacturer’s instructions.

### 2.6. PCR Amplification of Bacterial 16S rDNA

*Bifidobacterium*, *Clostridium*, *Lactobacillus*, *E. coli*, and *L. plantarum* primers were designed as previously described [23,24]. Universal primers for the invariant region of bacterial 16S rRNA were utilized for results normalization. PCR products were separated using electrophoresis on 2% agarose gel, stained with ethidium bromide, and quantified with Quantity One 1D software (BioRad, Hercules, CA, USA).

### 2.7. 16S rRNA Gene Amplification, Sequencing and Analysis

Performed as previously described [25]. Briefly, cecal bacterial DNA was extracted as defined by the manufacturer (PowerSoil DNA isolation kit, MoBio Laboratories Ltd., Carlsbad, CA, USA). Bacterial 16S rRNA gene sequences were PCR-amplified using the 515F-806R primers for the V4 hypervariable region of the 16S rRNA gene [7,25,26,27,28,29,30,31,32,33,34]. Detailed methodology is provided in the supplementary materials.

### 2.8. Glycogen Analysis

Glycogen content quantification in the pectoralis muscle and liver was performed as previously described [7,35]. Briefly, the frozen pectoralis muscle or liver samples were homogenized for 1 min in perchloric acid (8% *v*/*v*) on ice, centrifuged at 12,000× *g* for 15 min at room temperature, and the resulting supernatant was discarded. A measurement of 1 mL of petroleum ether was added, the petroleum ether fraction was discarded, and the lower layer of each sample was transferred to a 96-well plate containing iodine reagent (300 μL). Samples were read at 450 nm in a plate reader (Epoch, BioTek, VT, USA). The glycogen content was calculated using a standard curve.

### 2.9. Tissue Morphology Examination

Intestinal tissue morphometric assessment was performed as was previously described on duodenal sections [7,17,21]. Duodenum sections were fixed in 4% (*v*/*v*) buffered formaldehyde, dehydrated, cleared, and embedded in paraffin. Sections were cut (5 μm thickness) and positioned on glass slides, deparaffinized in xylene, rehydrated in ethanol, and stained with Alcian Blue/Periodic acid-Schiff. Villus height, villus width, crypt depth, Paneth cell number per crypt, Paneth cell width, goblet cell number, goblet cell diameter, goblet cell type within the villi, and goblet cell type within the crypts were assessed using a light microscope (CellSens Standard software, Olympus, Waltham, MA, USA). Five biological samples per treatment group (*n* = 5) and four segments for each biological sample were analyzed. Ten randomly selected villi and crypts were analyzed per segment and cell size measurements and counts were counted in ten randomly selected villi and/or crypts per segment (40 replicates per biological sample). Villus surface area was calculated using the following equation:

(1) $$Villus\;surface\;area=2\pi \;\times \;\frac{VW}{2}\times VL$$

where *VW* is the average of three measurements of villus width, and *VL* is the villus length.

### 2.10. Statistical Analysis

Results are shown as mean ± standard error, *n* = 6–12, in tables and heatmaps. Heatmaps were created in Microsoft Excel (Microsoft Corporation, Redmond, WA, USA) based on conditional formatting using color scales based on result means. Gene expression was normalized to 18S gene expression [22] and presented in arbitrary units (AU). To assess distribution normality, the Shapiro–Wilk test was used. Normally distributed results were analyzed by one-way ANOVA and Duncan post-hoc test. The Kruskal–Wallis test was utilized for non-parametric data. Differences were considered significant at *p* < 0.05. Statistical analyses were carried out using SPSS software (version 20.0, IBM, Armonk, NY, USA).

## 3. Results

### 3.1. Body Weight and Cecum Weight

The body weight of the 2.5% genistein group is significantly higher than the no-injection group (*p* < 0.05, Table 2). For cecum weights, the no-injection and H_2_O groups demonstrate significantly greater values when compared to the 5% inulin and 2.5% genistein groups (*p* < 0.05).

### 3.2. Hemoglobin and Glycogen Concentrations

Blood hemoglobin (Hb) levels in the 1.25% genistein group are significantly elevated compared to the no-injection, H_2_O, and 5% inulin groups (*p* < 0.05, Table 3). The blood hemoglobin of the 2.5% genistein group is higher than the no-injection group and significantly higher versus the H_2_O and 5% inulin groups. Among average glycogen, there were no significant differences between the genistein-treated and no-injection groups (*p* > 0.05).

### 3.3. Gene Expression of Fe, Zn, BBM Functionality, and Inflammation Related Proteins

#### 3.3.1. Fe-Related Proteins

**Figure 1:**
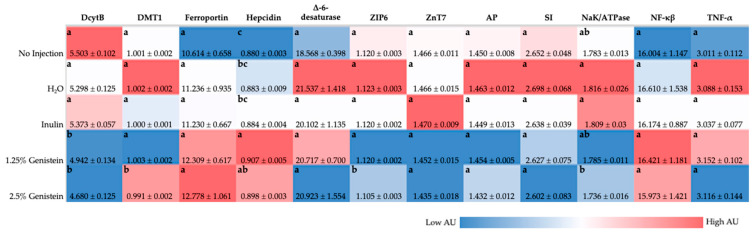
Effect of intraamniotic administration of genistein and controls on duodenal and liver (hepcidin) mRNA gene expression. Gene expression has been normalized to the 18S housekeeping gene and is in arbitrary units (AU). Values are presented as mean ± SEM, *n* = 6. ^a–c^ Per gene (in the same column), treatments groups not indicated by the same letter are significantly different (*p* < 0.05) according to one-way ANOVA with post-hoc Duncan test. DcytB, duodenal cytochrome b; DMT1, divalent metal transporter 1; ZIP6, zinc transport protein 6; ZnT7, zinc transporter 7; AP, amino peptidase; SI, sucrose isomaltase; NaK/ATPase, sodium, potassium and adenosine triphosphate; NF-κβ, nuclear factor kappa β subunit 1; TNF-α, tumor necrosis factor-α.

As depicted in Figure 1, gene expression of DMT1 is downregulated in the 2.5% genistein when compared to all other experimental groups (*p* < 0.05). DcytB was significantly downregulated (*p* < 0.05) in the genistein treatment groups compared to the no-injection, H_2_O, and inulin groups. Hepcidin was significantly upregulated (*p* < 0.05) with genistein exposure compared to the no-injection group. There were no significant differences in ferroportin expression between groups.

#### 3.3.2. Zn-Related Proteins

ZIP6 was significantly downregulated (*p* < 0.05) in the 2.5% genistein group compared to all other treatment groups (Figure 1). There were no significant differences in ZnT7 or Δ-6-desaturase expression between groups.

#### 3.3.3. Inflammatory Cytokines and BBM Functionality

No significant differences in gene expression of aminopeptidase (AP), sucrose isomaltase (SI), sodium, potassium, and adenosine triphosphate (NaK/ATPase) were found when comparing the treatment groups to the no-injection group (Figure 1). No significant differences in gene expression of nuclear transcription factor (NF-κβ) and tumor necrosis factor-α (TNF-α) between groups were found.

### 3.4. Morphometric Analysis of Duodenal Villi, Depth of Crypts, Goblet Cells, and Paneth Cells

The villus height, width, and surface area of the 2.5% genistein were significantly increased (*p* < 0.05) compared to the no-injection and H_2_O groups (Table 4). The 1.25% genistein group had significantly (*p* < 0.05) greater villus width than the no-injection and H_2_O groups. The 2.5% genistein group had significantly higher (*p* < 0.05) villus height, width and surface area compared to the 1.25% genistein.

The villi goblet cell diameter and total goblet cell number were significantly higher (*p* < 0.05) in the genistein-exposed groups than in the no-injection, H_2_O, and inulin groups (Table 5). More specifically, the acidic villi goblet cell count was significantly increased (*p* < 0.05) in the 1.25% genistein and 2.5% genistein groups relative to the 5% inulin, no-injection, and H_2_O control groups. The neutral villi goblet cell count of 1.25% genistein, 2.5% genistein, and 5% inulin groups were significantly higher (*p* < 0.05) compared with the no-injection and H_2_O injection controls, and the mixture villi goblet cells were significantly reduced (*p* < 0.05) with genistein exposure when compared with no-injection, H_2_O, and 5% inulin control groups.

As shown in Table 6, the crypt goblet cell diameter of the 1.25% genistein group was significantly larger (*p* < 0.05) than all control groups. The 2.5% genistein group had a significantly higher diameter than the 5% inulin group. Genistein exposure resulted in a significantly higher (*p* < 0.05) total crypt goblet cell count when compared with the no-injection and H_2_O groups. More specifically, the acidic crypt goblet cell count of both genistein treatment groups was significantly higher (*p* < 0.05) when compared with the no-injection, H_2_O, and inulin groups. Genistein exposure significantly reduced mixed crypt goblet cells (*p* < 0.05) compared with the no-injection, H_2_O, and inulin groups.

The number of crypt Paneth cells was significantly greater (*p* < 0.05) for the genistein treatment groups compared to the no-injection, H_2_O, and inulin groups (Table 7). The crypt depth for the genistein treatment groups was significantly lower (*p* < 0.05) compared to the H_2_O-injection group. The 1.25% genistein group had a significantly (*p* < 0.05) higher crypt Paneth cell diameter than the no-injection and 5% inulin groups.

### 3.5. Intestinal Content Bacterial Expression

**Figure 2:**
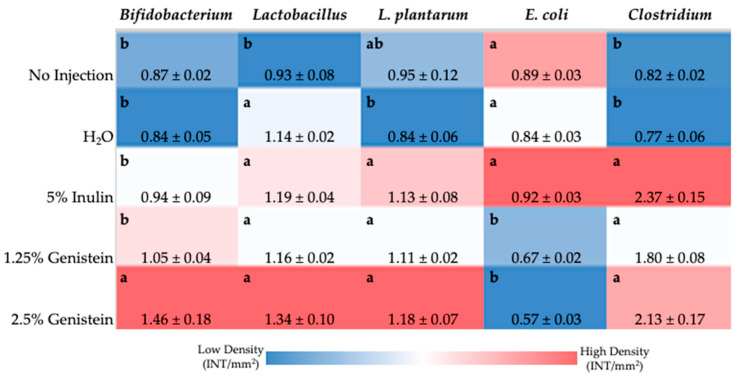
Effects of intraamniotic injections of genistein and the controls on duodenal genera and species-level bacterial populations. Values are presented as mean ± SEM, *n* = 5, as relative intensity of bands per mm^2^ of gel. ^a,b^ per bacterial category (in the same column), treatment groups that do not share any letters are significantly different (*p* < 0.05) according to one-way ANOVA with post-hoc Duncan test.

Figure 2 shows the duodenal genera and species-level bacterial populations. The relative abundance of *Bifidobacterium* spp., considered a probiotic bacteria, was significantly increased (*p* < 0.05) with 2.5% genistein exposure compared with all other treatment groups. *Lactobacillus* spp. relative abundance was significantly increased (*p* < 0.05) with genistein exposure compared to the no-injection control. *L. plantarum*, a probiotic bacteria associated with increased Fe absorption, was significantly increased (*p* < 0.05) in the genistein-exposed groups and 5% inulin control compared with the H_2_O-injected control. Genistein exposure significantly decreased (*p* < 0.05) the relative abundance of E. coli compared with all other experimental groups. *Clostridium* spp. relative abundance was significantly increased (*p* < 0.05) in the genistein-treated groups and 5% inulin control compared to the no-injection and H_2_O injection controls.

## 4. Discussion

In the current study, we have evaluated the effect of intraamniotic genistein administration on mineral transport, duodenal brush border membrane development and functionality, and intestinal microbiota. Although the ingestion of genistein has been associated with marked physiological changes associated with cancer and metabolic syndrome, further understanding of tissue-level effects associated with genistein exposure is needed [6,36,37]. Presently, there is a paucity of studies in the literature that directly measure the effects of genistein on the combination of mineral transport, BBM morphology or functionality, and intestinal microbiota.

The intraamniotic administration of genistein positively affected intestinal development, as demonstrated by increased enterocyte proliferation. The duodenal morphometric analysis demonstrated a significant (*p* < 0.05) dose-responsive effect of genistein treatment on increasing villus surface area versus the no-injection control (Table 4), indicative of improved digestive enzyme and absorptive capacity [7]. A significantly (*p* < 0.05) reduced crypt depth was observed with genistein administration when compared to the H_2_O injection control group (Table 7), which has been shown to be a marker of efficient tissue turnover and good condition of the gut [38]. The increase in villus surface area and reduction in crypt depth are in accordance with other genistein administration trials using the in vivo *Gallus gallus* model [39,40]. Additionally, increased proliferation in total villi and crypt goblet cells and an increase in the proportion of villi acidic and crypt acidic (*p* < 0.05) goblet cells were observed with genistein exposure compared to the no-injection and H_2_O injection controls (Table 5 and Table 6). This indicates increased synthesis and secretion of acidic luminal mucin by duodenal goblet cells [11,12]. The major goblet cell mucins in the small intestine are mucin 2 proteins, gel-forming secretory mucins that facilitate hydrolysis and absorption of nutrients [18,41,42,43]. In addition to serving as a protective intestinal epithelial barrier, this mucin (mucin 2) also functions as a habitat that supports probiotic populations and promotes epithelial cell function [44,45]. Taken as a whole, this demonstrates that the intraamniotic administration of genistein can positively modulate BBM development and functionality.

The intestinal microbiota of the *Gallus gallus* model is significantly and directly influenced by host genetics, environment, and diet [23,46]. At the phylum level, there is a significant resemblance between the gut microbiota of *Gallus gallus* and humans, with *Bacteroidetes*, *Firmicutes*, *Proteobacteria*, and *Actinobacteria* representing the dominant bacterial phyla [47]. Soy isoflavone treatment has been shown to alter intestinal bacterial populations in vivo, including increases in populations of SCFA-producing bacteria [1,36,48]. In the duodenum, the relative abundance of *Bifidobacterium* spp. considered a probiotic bacteria species, significantly increased with 2.5% genistein exposure compared with all other treatment groups (Figure 2). *Lactobacillus* spp. relative abundance was significantly increased with genistein exposure compared to the no-injection control. Further, linear discriminant analysis effect size (LefSe) analysis found that genistein treatment enriched bacterial pathways associated with *de novo* synthesis of vitamin B_12_ (Figure S1), where bacteria from the *Lactobacillus* genus represent a small number of bacteria known to encode the complete *de novo* biosynthetic pathway of vitamin B_12_ [49,50]. *L. plantarum*, a probiotic bacteria species associated with increased Fe absorption, was significantly increased in the genistein exposed-groups and 5% inulin control compared with the H_2_O-injected control [51]. *L. plantarum* produces glucosidases that can hydrolyze isoflavones (glycosides) into metabolites (aglycones) with increased antioxidant activity [52]. Increased populations of health-promoting bacteria, *Bifidobacterium* spp., *Lactobacillus* spp., and *L. plantarum*, resulting from genistein exposure, can be attributed to increased acidic mucin production [45,53,54]. Increased acidic mucin synthesis provides an environment conducive to the proliferation of these probiotic bacterial populations, which can be associated with an increased Paneth cell number per crypt and number of villi and crypt acidic goblet cells associated with genistein administration [45,53]. *Clostridium* spp. was significantly increased in the genistein-treated groups, and butyrate-producing (SCFA) bacteria, such as *Roseburia* spp. and *E. hallii* from *Clostridium* cluster XIVa, have previously been observed to be increased with genistein exposure in vitro [4]. The increase in *Lactobacillus* spp., *Bifidobacterium* spp., and *Clostridium* spp. abundance may further contribute to increased mineral bioavailability as these genera house SCFA-producing species, where SCFAs reduce the intestinal pH and thus may increase mineral (Fe and Zn) solubility and absorption [7,18,55].

Our previous research suggested soy isoflavone (daidzein) intraamniotic administration has the potential to improve dietary Fe bioavailability [19]. In our current study, BBM gene expression analysis (Figure 1) demonstrated that genistein downregulated DMT1 (transports Fe^2+^ into duodenal enterocyte) and DcytB (reduces Fe^3+^ to Fe^2+^) and upregulated ferroportin (transports Fe^2+^ into blood) and hepcidin (binds to ferroportin, causes ferroportin internalization and degradation), relative to the control group, though these results were not necessarily dose-dependent or significant [56,57,58,59]. Based on protein functionalities in Fe sufficient or excess scenarios, it is expected that DcytB, DMT1, and ferroportin would be downregulated, whereas hepcidin would be upregulated [57,58,60,61,62,63]. Though upregulation of ferroportin has previously been associated with Fe deficiency, genistein treatment was found to upregulate ferroportin expression in glial cells through estrogen receptor ß-dependent p38 MAPK activation, independent of Fe status [9,63]. Genistein administration has been shown to upregulate hepcidin expression, directly influencing ferroportin expression in in vivo and in vitro liver cell models [10]. Blood Hb levels were increased with genistein administration compared with the controls, which, taken together with Fe gene expression analysis, may indicate Fe status was improved by genistein administration. Genistein exposure resulted in ZIP6 (imports zinc across cell membrane) downregulation in comparison with the no-injection control, potentially indicative of improved zinc status with genistein administration [64,65], or could be associated with estrogenic effects of soy isoflavones, where ZIP6 expression was found to be modulated with anti-estrogen treatment in breast cells [66,67]. Although Zn absorption occurs in the duodenum, it has been suggested that the ileum is the leading site of Zn absorption in *Gallus gallus* [68], where future studies should focus on the Zn-transporter gene expression in the ileum to further understand the effects of genistein administration on Zn transport and absorption. Overall, alterations in mineral transport and hemoglobin concentration associated with improvements in mineral status can potentially be attributed to the combination of increased bacterial production of SCFA and increased proportion of acidic goblet cells associated with genistein exposure, resulting in a lowered intestinal pH and increased mineral solubility, thus improving mineral absorption [12,18,69].

Increases in body weight were observed in a dose-dependent manner compared with the controls, with the 2.5% genistein treatment group being significantly higher (*p* < 0.05) than the no-injection control (Table 2). Given the short exposure time, a significant increase in body weight is unexpected, but when taken with improved Fe status and BBM development, and given that the in vivo *Gallus gallus* model is sensitive to dietary Fe and Zn deficiencies [55,70], a significant increase in body weight confirms the positive developmental effects related to genistein exposure [71]. Additional studies are warranted to assess shifts in mineral status, intestinal functionality and development, and intestinal microbiota post-hatch and during a long-term feeding trial associated with genistein consumption.

## 5. Conclusions

This present study demonstrates intraamniotic administration of genistein improved brush border membrane functionality through improvements in villus architecture, goblet cell expansion, and related mucin production. Additionally, increases in the relative abundance of bacterial populations associated with SCFA production were found. Consequently, the combination of these factors contributed to alterations in the relative expression of various duodenal and hepatic proteins responsible for mineral absorption and transport associated with improved Fe status. Given these findings, genistein represents a promising plant bioactive and should be further evaluated in long-term animal and controlled human efficacy trials.

## Figures and Tables

**Table 1:** Sequences of primers used in this study.

Analyte	Forward Primer (5′–3′)	Reverse Primer (3′–5′)	Base Pair	GI Identifier
Iron Metabolism				
DcytB	CATGTGCATTCTCTTCCAAAGTC	CTCCTTGGTGACCGCATTAT	103	20380692
DMT1	TTGATTCAGAGCCTCCCATTAG	GCGAGGAGTAGGCTTGTATTT	101	206597489
Ferroportin	CTCAGCAATCACTGGCATCA	ACTGGGCAACTCCAGAAATAAG	98	423984
Hepcidin	AGACGACAATGCAGACTAACC	CTGCAGCAATCCCACATTTC	132	SAMN08056490
Zinc Metabolism				
Δ-6-desaturase	GGCGAAAGTCAGCCTATTGA	AGGTGGGAAGATGAGGAAGA	93	261865208
ZIP6	GCTACTGGGTAATGGTGAAGAA	GCTGTGCCAGAACTGTAGAA	380	66735072
ZnT7	GGAAGATGTCAGGATGGTTCA	CGAAGGACAAATTGAGGCAAAG	87	56555152
BBM Functionality				
AP	CGTCAGCCAGTTTGACTATGTA	CTCTCAAAGAAGCTGAGGATGG	138	45382360
SI	CCAGCAATGCCAGCATATTG	CGGTTTCTCCTTACCACTTCTT	95	2246388
NaK/ATPase	CCTTGGAGGTTTCTTCACCTATT	GGTCATCCCACTGAAGTCTAATC	92	14330321
Inflammatory Response				
NF-κβ	CACAGCTGGAGGGAAGTAAAT	TTGAGTAAGGAAGTGAGGTTGAG	100	2130627
TNF-α	GACAGCCTATGCCAACAAGTA	TTACAGGAAGGGCAACTCATC	109	53854909
18S	GCAAGACGAACTAAAGCGAAAG	TCGGAACTACGACGGTATCT	100	7262899

DcytB, duodenal cytochrome b; DMT1, divalent metal transporter 1; ZIP6, zinc transport protein 6; ZnT7, Zinc transporter 7; AP, amino peptidase; SI, Sucrose isomaltase; NaK/ATPase, Sodium, Potassium and adenosine triphosphate; NF-κβ, nuclear factor kappa β subunit 1; TNF-α, tumor necrosis factor-α.

**Table 2:** Effect of genistein exposure on body weight and cecum weight ^1^.

Treatment Group	Average Body Weight (g)	Average Cecum Weight (g)
No Injection	43.23 ± 1.44 ^b^	0.60 ± 0.05 ^a^
H_2_O	44.62 ± 1.43 ^ab^	0.59 ± 0.05 ^a^
5% Inulin	46.04 ± 1.18 ^ab^	0.43 ± 0.05 ^b^
1.25% Genistein	45.83 ± 0.99 ^ab^	0.50 ± 0.04 ^ab^
2.5% Genistein	47.69 ± 1.30 ^a^	0.44 ± 0.03 ^b^

^1^ Values are means ± SEM, *n* = 6. ^a,b^ Treatment groups not indicated by the same letter in the same column are significantly different (*p* < 0.05) according to one-way ANOVA with post-hoc Duncan test.

**Table 3:** Blood hemoglobin (Hb) concentrations (g/dL) and pectoral muscle glycogen concentrations (mg/g) following genistein exposure ^1^.

Treatment Group	Average Hb (g/dL)	Average Glycogen (mg/g)
No Injection	10.10 ± 2.40 ^bc^	0.019 ± 0.005 ^a^
H_2_O	9.68 ± 2.50 ^c^	0.014 ± 0.003 ^a^
5% Inulin	9.56 ± 0.92 ^c^	0.002 ± 0.001 ^b^
1.25% Genistein	14.98 ± 0.45 ^a^	0.008 ± 0.003 ^ab^
2.5% Genistein	14.23 ± 0.79 ^ab^	0.015 ± 0.004 ^a^

^1^ Values are the means ± SEM, *n* = 6–12. ^a–c^ Treatment groups not indicated by the same letter in the same column are significantly different (*p* < 0.05) according to one-way ANOVA with post-hoc Duncan test.

**Table 4:** Effects of genistein intraamniotic administration on duodenal small intestinal villus ^1^.

Treatment Group	Villus Height (µm)	Villus Width (µm)	Villus Surface Area (µm^2^)
No Injection	201.18 ± 4.94 ^b^	33.73 ± 0.67 ^e^	112.51 ± 4.28 ^d^
H_2_O	204.74 ± 4.52 ^b^	41.92 ± 1.01 ^d^	143.33 ± 5.27 ^c^
5% Inulin	246.64 ± 5.14 ^a^	50.98 ± 1.03 ^a^	206.92 ± 6.37 ^a^
1.25% Genistein	204.18 ± 3.73 ^b^	44.51 ± 0.86 ^c^	146.97 ± 4.55 ^c^
2.5% Genistein	238.22 ± 3.17 ^a^	48.27 ± 0.87 ^b^	184.13 ± 4.66 ^b^

^1^ Values are presented as mean ± SEM, *n* = 5. ^a–e^ Treatment groups not indicated by the same letter in the same column are significantly different (*p* < 0.05) according to one-way ANOVA with post-hoc Duncan test.

**Table 5:** Effects of genistein intraamniotic administration on villi goblet cells ^1^.

Treatment Group	Villi Goblet Cell Diameter (µm)	Villi Goblet Cell Number (Unit)			
		Acidic	Neutral	Mixture	Total
No Injection	2.86 ± 0.02 ^d^	13.59 ± 0.39 ^d^	0.01 ± 0.01 ^c^*	3.50 ± 0.23 ^c^	17.09 ± 0.49 ^d^
H_2_O	3.11 ± 0.03 ^c^	15.03 ± 0.39 ^c^	0.01 ± 0.01 ^c^*	5.76 ± 0.30 ^b^	20.80 ± 0.47 ^c^
5% Inulin	2.74 ± 0.03 ^e^	16.39 ± 0.54 ^c^	0.10 ± 0.02 ^b^*	6.53 ± 0.30 ^a^	23.02 ± 0.60 ^b^
1.25% Genistein	3.41 ± 0.03 ^b^	23.49 ± 0.67 ^a^	0.09 ± 0.03 ^b^*	1.69 ± 0.13 ^e^	25.26 ± 0.67 ^a^
2.5% Genistein	3.50 ± 0.03 ^a^	22.01 ± 0.51 ^b^	0.19 ± 0.04 ^a^*	2.70 ± 0.16 ^d^	24.89 ± 0.54 ^a^

^1^ Values are presented as mean ± SEM, *n* = 5. ^a–e^ Treatment groups not indicated by the same letter in the same column are significantly different (*p* < 0.05) according to one-way ANOVA with post-hoc Duncan test. ^a^*^–c^* Treatment groups indicated are significantly different (*p* < 0.05) based on Kruskal–Wallis.

**Table 6:** Effects of genistein intraamniotic administration on crypt goblet cells ^1^.

Treatment Group	Crypt Goblet Cell Diameter (µm)	Crypt Goblet Cell Number (Unit)			
		Acidic	Neutral	Mixture	Total
No Injection	2.68 ± 0.02 ^b^	5.46 ± 0.18 ^d^	0.00 ± 0.00 ^a^	1.49 ± 0.09 ^b^	6.95 ± 0.21 ^d^
H_2_O	2.65 ± 0.02 ^b^	6.07 ± 0.18 ^c^	0.00 ± 0.00 ^a^	1.76 ± 0.08 ^a^	7.83 ± 0.19 ^c^
5% Inulin	2.51 ± 0.02 ^c^	7.97 ± 0.16 ^b^	0.00 ± 0.00 ^a^	1.19 ± 0.08 ^c^	9.15 ± 0.16 ^b^
1.25% Genistein	2.89 ± 0.02 ^a^	8.51 ± 0.14 ^a^	0.00 ± 0.00 ^a^	0.74 ± 0.06 ^d^	9.25 ± 0.14 ^ab^
2.5% Genistein	2.63 ± 0.02 ^b^	8.81 ± 0.19 ^a^	0.00 ± 0.00 ^a^	0.88 ± 0.06 ^d^	9.68 ± 0.19 ^a^

^1^ Values are presented as mean ± SEM, *n* = 5. ^a–d^ treatment groups not indicated by the same letter in the same column are significantly different (*p* < 0.05) according to one-way ANOVA with post-hoc Duncan test.

**Table 7:** Effects of genistein intraamniotic administration on crypt depth and Paneth cells ^1^.

Treatment Group	Crypt Depth (µm)	# Crypt Paneth Cells	Crypt Paneth cell Diameter (µm)
No Injection	22.45 ± 0.39 ^d^	1.48 ± 0.05 ^d^	1.67 ± 0.03 ^b^
H_2_O	39.07 ± 0.80 ^a^	2.46 ± 0.11 ^c^	1.82 ± 0.04 ^a^
5% Inulin	35.00 ± 0.43 ^b^	2.56 ± 0.09 ^c^	1.68 ± 0.03 ^b^
1.25% Genistein	26.36 ± 0.46 ^c^	2.92 ± 0.10 ^b^	1.78 ± 0.04 ^a^
2.5% Genistein	23.65 ± 0.46 ^d^	3.24 ± 0.11 ^a^	1.65 ± 0.03 ^b^

^1^ Values are the means ± SEM, *n* = 5. ^a–d^ treatment groups not indicated by the same letter in the same column are significantly different (*p* < 0.05) according to one-way ANOVA with post-hoc Duncan test. The # symbol refers to the number of Paneth cells.

## Data Availability

Data available upon reasonable request.

## Supplementary Materials

The following supporting information can be downloaded at: https://www.mdpi.com/article/10.3390/nu14173473/s1, Figure S1: Bacterial pathways identified by the LEfSe method with the greatest differences in the control, 5% inulin, and genistein treated groups.
