# Supplementary material for: Intraamniotic Administration (Gallus gallus) of Genistein Alters Mineral Transport, Intestinal Morphology, and Gut Microbiota
Source: Nutrients. 2022 Aug 24;14(17):3473. doi: 10.3390/nu14173473 (PMC9458084; doi:10.3390/nu14173473)
Supplement: Supplementary file 1 [file nutrients-14-03473-s001.zip › nutrients-1866527-supplementary.pdf]

Intraamniotic Administration (*Gallus gallus*) of Genistein Alters Trace Mineral Transport, Intestinal Morphology, and Gut Microbiota

Jacquelyn Cheng [1], Nikolai Kolba [1], Philip Sisser [2], Sondra Turjeman[3], Carmel Even [3], Omry Koren [3] and Elad Tako [1,*]

[1] Department of Food Science, Cornell University, Stocking Hall, Ithaca, NY 14853, USA
[2] Department of Environment & Sustainability, Cornell University, Kennedy Hall, Ithaca, NY 14853, USA
[3] Azrieli Faculty of Medicine, Bar-Ilan University, 1311502 Safed, Israel
* Correspondence: et79@cornell.edu; Tel.: +1-607-255-0884

Materials and Methods

16S rRNA Gene Sequence Analysis

Microbial genomic DNA was extracted from cecal samples using the PowerSoil DNA isolation kit (MoBio Laboratories Ltd., Carlsbad, CA, USA), as described by the manufacturer (Control: n = 6, 5% inulin: n = 3, and genistein: n = 5). Bacterial 16S rRNA gene sequences were PCR-amplified from each sample using the 515F-806R primers for the V4 hypervariable region of the 16S rRNA gene, including 12-base barcodes, as previously published [1]. PCR procedure reactions consisted of 25 µL Primestar max PCR mix (Takara Kusatsu, Shiga, Japan), 2 µM of each primer, 17 µL of ultra-pure water, and 4 µL DNA template. Reaction conditions consisted of an initial denaturing step for 3 min at 95°C followed by 30 cycles of 10s at 98°C, 5s at 55°C, 20s at 72°C, and final elongation at 72°C for 1 min. PCR products were then purified with Ampure magnetic purification beads (Beckman Coulter, Atlanta, GA, USA) and quantified using a Quant-iT PicoGreen dsDNA quantitation kit (Invitrogen, Carlsbad, CA, USA). Equimolar ratios of total samples were pooled and sequenced at the Azrieli Faculty of Medicine of the Bar Ilan University (Safed, Israel) using an Illumina MiSeq Sequencer (Illumina, Inc., Madison, WI, USA).

16S rRNA gene sequence analysis was performed as previously described [2]. For quality filtering of raw data, sequences with Phred < 20, or shorter than 75% of the expected length were discarded, as well as sequences containing primer mismatches, uncorrectable barcodes, ambiguous bases, or homopolymer runs in excess of 6 bases. The sequences that passed the quality filters were analyzed using the QIIME software package [3]. Sequences were classified taxonomically using the Greengenes (GG) reference database at a confidence threshold of 80% [4]. The GG taxonomies were used to generate summaries of the taxonomic distributions of OTUs across different levels (phylum, order, family, and genus). To standardize sequence counts across samples with uneven sampling, we randomly selected 22,450 sequences per sample (rarefaction) and used this as a basis to compare abundances of OTUs across samples. For phylogenetic tree-based analyses, each OTU was represented by a single sequence that was aligned using PyNAST [5]. A phylogenetic tree was built with Fast-Tree [6] and used for estimates of α-diversity (within sample diversity, using Faith's phylogenetic diversity [7]) and β-diversity (between sample diversity, using unweighted and weighted UniFrac [8]). For PD measurements, means and standard errors for given categories were calculated from 100 iterations using a rarefaction of 16,837 sequences per sample. Metagenome functional predictive analysis was carried out using PICRUSt software predictive functional profiling of microbial communities, using 16S rRNA marker gene sequences [9]. Briefly, OTU abundance was normalized by 16S rRNA gene copy number, identified, and compared to a phylogenetic reference tree using the Greengenes database, and was assigned functional traits and abundance based on known genomes and prediction using the Kyoto Encyclopedia of Genes and Genomes (KEGG) [9].

Microbiome Statistical Analysis

Statistically significant p-values associated with microbial clades and functions identified by Linear discriminant analysis Effect Size (LefSe) were corrected for multiple comparisons using the Benjamini–Hochberg false discovery rate (FDR) correction.

Results

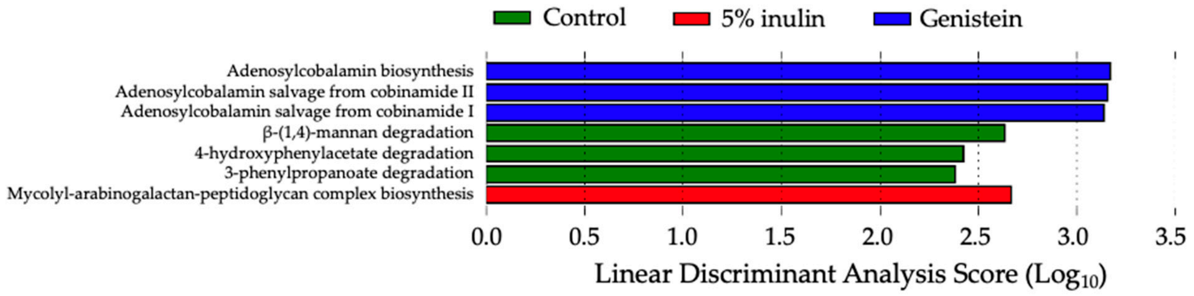

Figure S1. LEfSe method identifying the bacterial pathways with the greatest differences in the control, 5% inulin, and genistein treated groups.

The LEfSe method identified enriched bacterial pathways associated with biosynthesis of vitamin B12 with genistein exposure. In the control, pathways associated with mannan, amino acid, and phenolic compound degradation were enriched. In the 5% inulin group, a pathway associated with cell wall biosynthesis was enriched.

References

1. Carboni, J.; Reed, S.; Kolba, N.; Eshel, A.; Koren, O.; Tako, E. Alterations in the Intestinal Morphology, Gut Microbiota, and Trace Mineral Status Following Intra-Amniotic Administration (Gallus gallus) of Teff (Eragrostis tef) Seed Extracts. *Nutrients* **2020**, *12*, doi:10.3390/nu12103020.
2. Reed, S.; Neuman, H.; Glahn, R.P.; Koren, O.; Tako, E. Characterizing the gut (Gallus gallus) microbiota following the consumption of an iron biofortified Rwandan cream seeded carioca (Phaseolus Vulgaris L.) bean-based diet. *PLoS One* **2017**, *12*, e0182431, doi:10.1371/journal.pone.0182431.
3. Caporaso, J.G.; Kuczynski, J.; Stombaugh, J.; Bittinger, K.; Bushman, F.D.; Costello, E.K.; Fierer, N.; Pena, A.G.; Goodrich, J.K.; Gordon, J.I., et al. QIIME allows analysis of high-throughput community sequencing data. *Nat Methods* **2010**, *7*, 335-336, doi:10.1038/nmeth.f.303.
4. McDonald, D.; Price, M.N.; Goodrich, J.; Nawrocki, E.P.; DeSantis, T.Z.; Probst, A.; Andersen, G.L.; Knight, R.; Hugenholtz, P. An improved Greengenes taxonomy with explicit ranks for ecological and evolutionary analyses of bacteria and archaea. *ISME J* **2012**, *6*, 610-618, doi:10.1038/ismej.2011.139.
5. Caporaso, J.G.; Bittinger, K.; Bushman, F.D.; DeSantis, T.Z.; Andersen, G.L.; Knight, R. PyNAST: A flexible tool for aligning sequences to a template alignment. *Bioinformatics* **2010**, *26*, 266-267.
6. Price, M.N.; Dehal, P.S.; Arkin, A.P. FastTree: computing large minimum evolution trees with profiles instead of a distance matrix. *Mol Biol Evol* **2009**, *26*, 1641-1650, doi:10.1093/molbev/msp077.
7. Faith, D. Conservation evaluation and phylogenetic diversity. *Biol Conserv* **1992**, *61*, 1-10.
8. Lozupone, C.; Knight, R. UniFrac: a new phylogenetic method for comparing microbial communities. *Appl Environ Microbiol* **2005**, *71*, 8228-8235, doi:10.1128/AEM.71.12.8228-8235.2005.
9. Langille, M.G.; Zaneveld, J.; Caporaso, J.G.; McDonald, D.; Knights, D.; Reyes, J.A.; Clemente, J.C.; Burkepile, D.E.; Vega Thurber, R.L.; Knight, R., et al. Predictive functional profiling of microbial communities using 16S rRNA marker gene sequences. *Nat Biotechnol* **2013**, *31*, 814-821, doi:10.1038/nbt.2676.
